# Indirect Detection of an Epitope-Specific Response to HIV-1 gp120 Immunization in Human Subjects

**DOI:** 10.1371/journal.pone.0027279

**Published:** 2011-11-04

**Authors:** Evgeny Shmelkov, Arthur Nadas, James Swetnam, Susan Zolla-Pazner, Timothy Cardozo

**Affiliations:** 1 Department of Pharmacology, New York University School of Medicine (NYUSoM), New York, New York, United States of America; 2 Department of Environmental Medicine, New York University School of Medicine (NYUSoM), New York, New York, United States of America; 3 Department of Pathology, New York University School of Medicine (NYUSoM), New York, New York, United States of America; 4 Veterans Affairs Medical Center, New York, New York, United States of America; University of Delhi, India

## Abstract

A specific response of human serum neutralizing antibodies (nAb) to a conformational epitope as a result of vaccination of human subjects with the surface envelope glycoprotein (gp120) of HIV-1 has not previously been documented. Here, we used computational analysis to assess the epitope-specific responses of human subjects, which were immunized with recombinant gp120 immunogens in the VAX003 and VAX004 clinical trials. Our computational methodology—a variation of sieve analysis—compares the occurrence of specific nAb targeted conformational 3D epitopes on viruses from infected individuals who received vaccination to the occurrence of matched epitopes in the viruses infecting placebo subjects. We specifically studied seven crystallographically defined nAb targeted conformational epitopes in the V3 loop, an immunogenic region of gp120. Of the six epitopes present in the immunogens and targeted by known monoclonal neutralizing antibodies, only the one targeted by the anti-V3 nAb 2219 exhibited a significant reduction in occurrence in vaccinated subjects compared to the placebo group. This difference occurred only in the VAX003 Thailand cohort. No difference was seen between vaccinated and placebo groups for the occurrence of an epitope that was not present in the immunogen. Thus, it can be theorized that a specific 2219-like human neutralizing antibody immune response to AIDSVAX immunization occurred in the VAX003 cohort, and that this response protected subjects from a narrow subset of HIV-1 viruses circulating in Thailand in the 1990s and bearing the conformational epitope targeted by the neutralizing antibody 2219.

## Introduction

In 1998 and 1999, two Phase III human clinical trials were conducted by VaxGen (VaxGen Inc, South San Francisco, CA) to test the efficacy of the AIDSVAX™ HIV vaccine. The AIDSVAX vaccine consisted of a bivalent immunogen derived from the recombinant envelope glycoprotein gp120 of HIV-1 subtypes B and E (strains MN and A244) in VAX003, and recombinant gp120 molecules from subtype B (strains MN and GNE8) in VAX004 [1], [2]. The choice of the HIV-1 strains was made based on phylogenetic analysis to cover the majority of HIV strains present in the regions where the clinical trials were conducted [3]–[7]. In the VAX003 and VAX004 randomized double-blind, placebo-controlled Phase III clinical trials, a total of 7963 volunteers were enrolled, and 611 of them were infected with HIV-1 during the study [1], [2]. The human subjects in the VAX003 and VAX004 trials thus represent existing, well-characterized placebo-controlled vaccination research cohorts.

AIDSVAX vaccination failed to broadly protect the overall study population (6.7% infection rate in the vaccinees, compared to 7.0% in the placebo group, p>0.1), although certain subgroups (non-Hispanic ethnic minorities, i.e. Asians and Africans) may have experienced protection [1], [2], [8]. Protection, if it occurred, may have correlated with higher titers of antibodies in those groups to the matched viral strains, MN, GNE8, or A244, but the gp120 amino acid sequences of the infecting viruses were substantially different from those in the immunogens [9], making the detection of a protective effect by traditional means extremely difficult.

The third variable loop (V3 loop) of the HIV-1 surface envelope glycoprotein (gp120) is an immunogenic region of the viral envelope [10]–[12]. The V3 loop is known to contain epitopes that induce both broadly and narrowly cross-reactive neutralizing antibodies [12]–[16]. After the VAX004 clinical trial, only one linear, one-dimensional (1D), sequence-defined V3 loop region was evaluated as a putative antibody-targeted viral epitope – the GPGRAF motif, presented in the V3 loop of the gp120's from MN and GNE8 strains. The presence of this sequence motif in infecting viruses did not affect the degree of correlation between antibody levels or HIV-1 incidence [1], [9]. No crystal structure exists of a monoclonal antibody specific for the entire GPGRAF sequence, and a linear peptide like GPGRAF present in an immunogen may give rise to many nAbs with diverse binding modes overlapping within the GPGRAF peptide. Furthermore, the activities of nAbs that engage epitopes not completely included in the GPGRAF fragment but encompassing amino acid atoms in nearby areas of the V3 loop crown were missed. Thus, the published study using the GPGRAF fragment [9] was not truly epitope-specific and could have detected only a fraction of the currently known V3 epitopes defined by 3D structures of V3-Ab crystallographic complexes.

HIV vaccine-induced immune responses that are undetectable by laboratory tests may be detected via “sieve” effects, wherein HIV acquisition is partially blocked (only certain viruses matched to the immune response are blocked). Prior attempts at HIV vaccine trial sieve analysis have only focused on linear T-cell epitopes, as defining conformational epitopes in linear DNA or amino acid sequences presents a significant technical challenge to traditional sieve analysis [17], [18]. We attempted to meet this challenge with a sieve analysis of the true conformational epitope-specific human nAb response to immunization with the AIDSVAX vaccines using a novel computational structural biology method for the detection of three-dimensionally (3D) -defined conformational epitopes in gp120. The three-dimensional conformational epitopes are projected into one-dimensional sequence space via sensitive and specific “signature motifs” defined using a panel of seven anti-V3 monoclonal antibodies' crystal structures [19], [20]. Briefly, in this method, a conformational 3D-epitope is defined by a number of sequentially-disparate but 3D space-clustered amino acids positions (a sequence motif representing the signature of the 3D conformational epitope: “signature motif”). The amino acids are restricted to those for which their side chains are engaged in buried atomic contacts within the 3D molecular surface of a monoclonal nAb in the crystallographic complex of the nAb with its cognate bound viral peptide [19], [20]. The resolving of the 3D conformational epitope shape bound by the specific monoclonal nAb into a simple sequence motif affords rapid and accurate classification of any library of gp120 viral sequences into those that contain the specific conformational 3D epitope and those that do not.

In the present study, we report application of this methodology in a novel form of sieve analysis [21], analyzing the library of sequences of the viruses infecting vaccine and placebo recipients in the VAX003 and VAX004 clinical trials (published by Global Solutions for Infectious Diseases (GSID)). Specifically, we classified the viruses infecting placebo and vaccinated patients into those that contain certain conformational epitopes and those that do not using the epitope motifs targeted by seven anti-V3-loop neutralizing monoclonal antibodies [20], [22]. The exploratory metric used was that one or more of the epitopes would show a lower rate of occurrence in viruses infecting vaccinated subjects as compared to viruses infecting placebo subjects, potentially indicating that the vaccine imposed immune selection pressure that reduced the occurrence of infection of the vaccinated subjects with viruses bearing those epitopes.

## Materials and Methods

### Ethics Statement

The only human subjects' data used in the current study were blinded data on HIV-1 gp120 sequences, which are publicly available through the GSID HIV Data Browser, therefore no IRB approval is required.

### Signature motifs for neutralization epitopes targeted by nAbs

**Figure 1:**
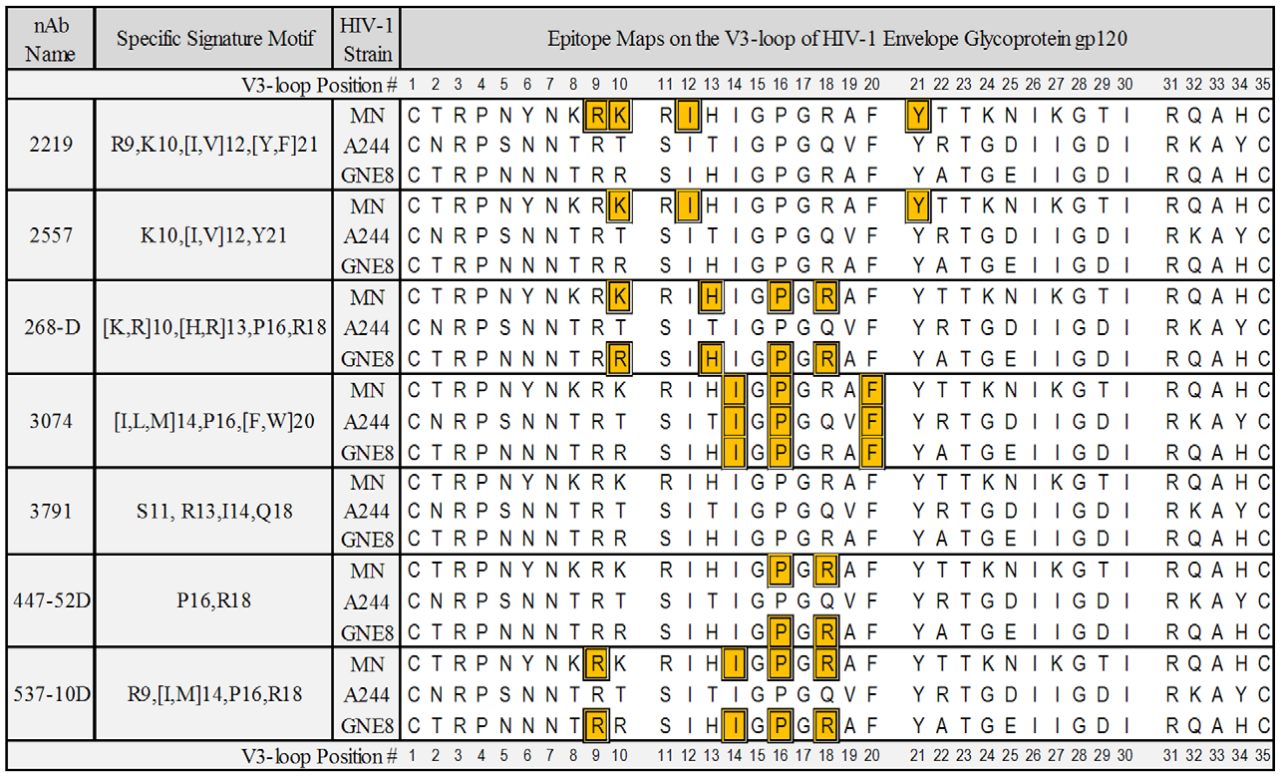
Library of signature motifs mapped to the V3 regions of AIDSVAX immunogens. Signature motifs recognized by each monoclonal antibody are shown in the second column: the numbers indicate the recognized positions in the V3 loop and the letters indicate the amino acid residues at each position required for recognition by that monoclonal antibody. Positions numbering is in the correspondence with the standard V3 loop numbering described elsewhere [19], [20]. Colored rectangles in the sequence column indicate the amino acid residues composing the signature motif in each of the gp120s. Sequences without colored rectangles do not contain a given motif.

Signature motifs for the epitopes of seven broadly neutralizing human monoclonal antibodies - 2219, 2557, 447-52D, 537-10D, 268-D, 3074 and 3791 [19], [20] were used for this study. Each signature motif targets an epitope in the V3 loop of gp120 (**Figure 1**).

### Vaccine immunogens

The sequences of the V3 loops of the recombinant gp120s used in AIDSVAX vaccines (**Figure 1**) were derived from GSID Data Browser (GSID HIV Data Browser; URL: http://www.gsid.org/gsid_hiv_data_browser.html; 9/2009).

### Glycoprotein gp120 sequences of HIV-1 infected patients

All V3 sequences of infecting viruses were obtained from the GSID Data Browser. A total of 382 volunteers were infected during VAX004 and 229 volunteers were infected during VAX003 Phase III clinical trials. 14 (VAX004) and 18 (VAX003) of the subjects had HIV-1 infection detected at month 0 and thus were excluded from future studies. For 32 volunteers infected during VAX004 and 14 volunteers infected during VAX003, no gp120 sequencing data are available. Therefore as sequence-based epitope characterization could not be performed on these subjects, they also were not included in the analysis. Thus, in the current study viral sequences from 533 (336 from VAX004, and 197 from VAX003) human subjects were analyzed. In both trials the average number of days between the estimated date of infection and the date of sample collection for ‘placebo’ and ‘vaccine’ cohorts was nearly the same (in VAX003: 128 days for ‘placebo’, 134 days for ‘vaccine’; in VAX004: 108 days for ‘placebo’, 111 days for ‘vaccine’). According to the trial protocol, at the evaluation time point in VAX004 exactly 3 randomly selected clones were sequenced from each infected volunteer. In VAX003 on average 3 randomly selected clones per patient were sequenced (1 patient with 2 clones, 184 patients with 3 clones each, 8 patients with 4 clones each, and 4 patients with 6 sequenced clones each). Importantly, the average number of clones per patient in both ‘placebo’ and ‘vaccine’ cohorts of VAX003 was equal. In the current study we considered a given human subject to be infected with an epitope-decorated virus if at least one of the collected sequences contains that epitope.

### Epitope mapping and analysis

Mapping of specific neutralization epitopes in the viruses infecting the study subjects was performed by searching the sequences of each of the infecting viruses with each signature motif listed in **Figure 1**. For the study, we asked the exploratory question: could any of the nAbs elicited by AIDSVAX immunization possibly protect against infection by the narrow subset of viruses containing the nAb's specific cognate signature motifs? Specifically, the study was designed to address that question by comparing the infection counts in vaccinated and placebo groups (i.e. comparing the number of viruses infecting vaccinees and containing signature motif “X” to the number of viruses infecting placebo subjects containing the same signature motif “X”) with the expectation that the counts in the vaccinated group would be lower than in the placebo group. P-values for each comparison were estimated using a right-tailed Fisher Exact test (or one-tailed hypergeometric test).

## Results

### The V3 loop signature motifs present in the vaccine

Signature motifs specific for the conformational epitopes targeted by seven different anti-V3 loop neutralizing human monoclonal antibodies were identified in our previous studies. The presence of these motifs, and their associated conformational epitopes, in the V3 loop sequences of the recombinant gp120 proteins used as immunogens during the AIDSVAX clinical trials are shown in **Figure 1**. The subtype B HIV-1 strain MN, used in both VAX003 and VAX004 clinical trials, contains six of the conformational epitopes — those targeted by monoclonal antibodies 268-D, 3074, 447-52D, 537-10D, 2219 and 2557. The second subtype B HIV-1 gp120 from GNE8, which was tested in the VAX004 clinical trial in North America and Europe, contains only four of the seven conformational epitopes, those targeted by monoclonal antibodies 268-D, 3074, 447-52D and 537-10D. The subtype E HIV-1 strain utilized in the AIDSVAX vaccine during the VAX003 clinical trial in Thailand, A244, has only one of seven conformational epitopes, that recognized by monoclonal antibody 3074. None of these three protein immunogens has the signature motif for the conformational epitope targeted by monoclonal antibody 3791, so the infection counts of viruses with this motif serves as an important internal control: there should be no difference in counts between the two groups since no response could have occurred to an epitope that was not present in the immunogen.

### The occurrence of signature motifs in sequences of viruses recorded from the AIDSVAX trials

We constructed a dataset containing all the V3 loop sequences of gp120s from the viruses listed in the GSID Data Browser (see “Materials and Methods”) representing all of the viruses identified to have infected the clinical trial subjects in VAX003 and VAX004. The dataset was divided into VAX003 and VAX004 groups of study subjects. Each of these two groups was separated into those who received vaccine and those who received placebo injections. The counts of viruses with each of the 7 signature motifs were recorded and compared as described in the Methods section. Lower counts with a specific signature motif in viruses infecting vaccinated subjects compared to the counts with that same motif in viruses infecting placebo subjects could be interpreted as evidence of the occurrence of a conformational-epitope-specific immune antibody response in vaccinees which prevented infection or after-infection outgrowth of viruses bearing the conformational epitope represented by that motif. The results of this comparison are summarized in the **Table 1**. Notably, the count for viruses containing the 2219 signature motif was significantly (p = 0.033) lower in the vaccinees in the Thailand VAX003 cohort as compared to the count of viruses containing the 2219 motif in the placebo group (**Table 1**). The infection counts for the signature motif for the epitope targeted by monoclonal antibody 2557 are only slightly different from those for the motif 2219 (**Table 1**). However, as the 2557-specific motif is a subset of that for 2219 (**Figure 1**), the results for 2557 and 2219 might be interdependent. Thus, most likely, observed reduction in the counts for the 2557-specific motif is just an artifact conditioned by the similarity of the epitope targeted by 2557 to the epitope targeted by 2219. The infection counts for viruses exhibiting other signature motifs were approximately equivalent (accounting for the difference in sample size) in vaccinees and placebo subjects. The infection counts for viruses exhibiting the 3791 epitope, which was not present in the immunogen, were also nearly identical between the vaccinees and placebo subjects in both trials.

**Table 1:** Screening for the signature motifs in datasets of gp120 sequences of HIV-1 strains infecting volunteers during AIDSVAX Phase III clinical trials.

AIDSVAX Trial ID:		VAX003			VAX004		
Infection Status	Viral sequence	Placebo	Vaccine	p-value	Placebo	Vaccine	p-value
Infected	268-D containing	1	2	0.879	33	105	1.000
Infected	447-52D containing	16	18	0.725	74	168	0.999
Infected	537-10D containing	9	12	0.828	64	142	0.986
Infected	2219 containing	23	12	0.033	85	156	0.588
Infected	2557 containing	21	13	0.099	75	153	0.936
Infected	3074 containing	84	82	0.488	103	181	0.275
Infected	3791 containing	9	9	0.606	1	1	0.584
Infected	Unknown	6	8	n/a	8	24	n/a
Infected	Total	105	106	n/a	127	241	n/a
Not Infected	Total	1143	1155	n/a	1675	3346	n/a

## Discussion

In previous studies [19], [20], we identified seven overlapping signature motifs specific for seven interspersed conformational neutralization epitopes present in the V3 loop crown. These motifs were defined as the critical residues recognized by various broadly neutralizing human anti-V3 monoclonal antibodies, and allowed us to identify the presence or absence of specific conformational epitopes in any HIV-1 virus [19], [20]. In the current study, we used this novel capability to identify the presence or absence of these epitopes in vaccine immunogens and in break-through viruses infecting vaccine and placebo recipients in the VAX003 and VAX004 Phase III clinical trials [1], [2].

Given the specificity of the sequence motifs for *neutralization* epitopes [19], [20], and given also the internal negative control of the 3791-targeted neutralization epitope, which worked as expected, we theorize that a protective neutralizing antibody response might occur in VAX003 human subjects in Thailand to the epitope recognized by monoclonal antibody 2219 as a result of immunization with the AIDSVAX B/E immunogen. However, it is likely that the hypothesized response was mediated by polyclonal nAbs similar to 2219 and other nAbs (e.g. 2557, or not tested in the study nAb), which have similar binding modes to 2219 and thus a similar signature motif as mAb 2219.

This finding is interesting in light of a previous study showing that the effective neutralization potential of the 2219 antibody (i.e. the cross-subtype breath after controlling for V3 epitope masking) is the highest among tested V3-loop specific nAbs [20], [22]. Anti-variable loop antibodies have historically been viewed with skepticism of their vaccine potential because the epitopes they target are either easily escaped by frequent mutations (e.g. only a narrow subset of viruses contains the epitope) or the epitopes, if present, are masked by glycans or other variable loops (epitope masking) [23]. Indeed, the prior study showed that different anti-V3 antibodies have widely variable breadths of occurrence of their targeted epitopes in circulating HIV viruses and widely variable levels of masking of those epitopes when present [20], [22]. One might expect that the only anti-V3 loop nAbs that have a chance of showing a protective effect in a clinical trial would be those that have the widest breadth of occurrence of their targeted epitopes in circulating viruses combined with the lowest level of masking of that epitope. Indeed, the conformational epitope targeted by nAb 2219 was shown to be the least masked (i.e. most accessible for nAbs) while occurring in 56% of circulating worldwide viruses, therefore having the highest effective neutralization potential among tested anti-V3 loop nAbs against circulating viruses [22].

If protection against the 2219-targeted conformational epitope actually occurred in the Thai population, the lack of a difference in the occurrence of the 2219 epitope between vaccine and placebo in the North American cohort suggests one of the following interpretations, or combinations thereof: a) that the AIDSVAX B/E immunogen, which was used exclusively in VAX003, confers a protective 2219 epitope-specific response, while AIDSVAX B/B, used in North America, does not; b) that AIDSVAX B/E only protects against viruses carrying the 2219 epitope in the population of HIV viruses circulating in Thailand (mainly subtype E) and not against the virus population infecting the US trial population (predominantly clade B, with many having V3 loops identical to the MN strain) [4], [6], [7]; c) that AIDSVAX B/E only protects against viruses carrying the 2219 epitope in the setting of non-sexual and/or heterosexual transmission, a possibly important factor given that the HIV risk factors of the North American cohort (MSM and heterosexual transmission) were indeed different from the Thailand cohort (IVDU and heterosexual transmission); or d) other undetermined host factors, such as differences in HLA class II repertoire. Interestingly, only AIDSVAX B/E was used in the recently successful RV144 HIV vaccine Phase III clinical trial in the Thailand population using a prime-boost vaccine regimen [24].

Although the overall VaxGen trial showed no broad protection, this study detected a statistically significant reduction of counts of the 2219-targeted conformational epitope in viruses infecting vaccinees as compared to viruses infecting placebo recipients in a randomized placebo-controlled blinded clinical trial. Although the most obvious hypothetical explanation of such difference is that vaccine-induced protection against viruses carrying the 2219-targeted conformational epitope have occurred, this is only a statistical result and other explanations are possible, including undetected bias in the trial design. One possible way to address that issue is to perform a follow-up experimental validation of the 2219-induced protection in uninfected Thai vaccinees. However, this is a computational study intended to convey to the community 1) a novel sieve analysis method that addresses the challenge of analyzing conformational antibody-targeted epitopes; and 2) a potentially high-impact epidemiological result, which was previously missed due to the unavailability of the recently published powerful conformational epitope classification method [17], [18]. It is also important to note, that the purpose of the presented statistical analysis was exploratory (hypothesis generation), not statistical hypothesis testing. Therefore, no correction for multiple testing was applied in order to avoid any false negative predictions.

In summary, in the present paper we describe a novel approach for computational profiling of the human antibody response based on counts in viral sequences of precisely defined signature motifs that characterize the 3D conformational neutralization epitopes targeted by specific anti-V3 neutralizing monoclonal antibodies. The approach potentially identified, by inference, for the first time a protective immune response to a specific HIV envelope conformational antibody-targeted epitope as a result of immunization in humans. Thus, the current work suggests that immunogens amplifying the elicitation of 2219-like (e.g., 2219 or 2557) antibodies might have potential value as HIV vaccine leads. In addition, this type of analysis could be critical for the interpretation of the data derived from prospective clinical trials of vaccines designed to induce protective antibodies.
